# Shining a spotlight on m6A and the vital role of RNA modification in endometrial cancer: a review

**DOI:** 10.3389/fgene.2023.1247309

**Published:** 2023-10-11

**Authors:** Zujian Jin, Jingjing Sheng, Yingying Hu, Yu Zhang, Xiaoxia Wang, Yiping Huang

**Affiliations:** ^1^ Department of Gynecology and Obstetrics, The Fourth Affiliated Hospital, Zhejiang Provincial Clinical Research Center for Obstetrics and Gynecology, Zhejiang University School of Medicine, Yiwu, Zhejiang, China; ^2^ Reproductive Medicine Center, School of Medicine, The Fourth Affiliated Hospital, Zhejiang University, Yiwu, Zhejiang, China

**Keywords:** RNA modification, endometrial cancer, N6-methyladenosine (m6A), 5-methylcytidine (m5c), RNA therapy

## Abstract

RNA modifications are mostly dynamically reversible post-transcriptional modifications, of which m6A is the most prevalent in eukaryotic mRNAs. A growing number of studies indicate that RNA modification can finely tune gene expression and modulate RNA metabolic homeostasis, which in turn affects the self-renewal, proliferation, apoptosis, migration, and invasion of tumor cells. Endometrial carcinoma (EC) is the most common gynecologic tumor in developed countries. Although it can be diagnosed early in the onset and have a preferable prognosis, some cases might develop and become metastatic or recurrent, with a worse prognosis. Fortunately, immunotherapy and targeted therapy are promising methods of treating endometrial cancer patients. Gene modifications may also contribute to these treatments, as is especially the case with recent developments of new targeted therapeutic genes and diagnostic biomarkers for EC, even though current findings on the relationship between RNA modification and EC are still very limited, especially m6A. For example, what is the elaborate mechanism by which RNA modification affects EC progression? Taking m6A modification as an example, what is the conversion mode of methylation and demethylation for RNAs, and how to achieve selective recognition of specific RNA? Understanding how they cope with various stimuli as part of *in vivo* and *in vitro* biological development, disease or tumor occurrence and development, and other processes is valuable and RNA modifications provide a distinctive insight into genetic information. The roles of these processes in coping with various stimuli, biological development, disease, or tumor development *in vivo* and *in vitro* are self-evident and may become a new direction for cancer in the future. In this review, we summarize the category, characteristics, and therapeutic precis of RNA modification, m6A in particular, with the purpose of seeking the systematic regulation axis related to RNA modification to provide a better solution for the treatment of EC.

## 1 Introduction

It is commonly believed that RNA is the vector that transmits genetic information from DNA to the machinery that synthesizes proteins. Aside from translation, RNA has a hand in many other essential biological processes such as genetic controlling, protein synthesis, degradation, and even catalysis of chemical reactions, which largely rely on intricate RNA modification and structure (Liu and Pan, 2015; Roundtree et al., 2017). In non-coding RNA species such as ribosomal RNA (rRNA) and transfer RNA (tRNA), chemical modifications have long been observed. In addition to mRNA and long non-coding RNAs (lncRNAs), an increasing number of RNA modifications have also been characterized. The rapid development of epigenomics research is manifested in the identification of RNA modifications and the in-depth exploration of the mechanism. It has been reported that more than 170 chemical modifications have been discovered in RNA, thus establishing a new layer of gene expression regulation referred to as the “epitranscriptome” (Barbieri and Kouzarides, 2020). Among the three major types of malignant tumors in the female reproductive system, endometrial cancer (Ec) is one of the most prevalent types in developed countries and the incidence rate is growing quickly (Siegel et al., 2023). Active surgery (Hysterectomy and bilateral salpingo-oophorectomy) are the mainstay treatments for EC, followed by adjuvant treatment based on histology and stage. Early diagnosis and treatment of EC are associated with better prognosis, but recurrent or primary metastatic ones are difficult to treat and have shorter median overall survival (OS) (Inoue et al., 2021). As of now, there are no endorsed focused treatments available for EC. It is crucial to gain more insight into epigenetic mechanisms to create alternative therapies for EC. Fortunately, a variety of methods and advanced detection tools are available to identify and increase the recognition of ribonucleoside modifications, both at a whole genome scale and specific nucleotide resolution, consequently, aside from their role in biology, RNA modifications can be used to improve RNA-based therapies, which are dramatically favorable for diagnosis and treatment for EC. We believe that in the near future, RNA therapy due to RNA modification will bring better opportunities for endometrial cancer patients.

## 2 Overview of RNA modification

**FIGURE 1:**
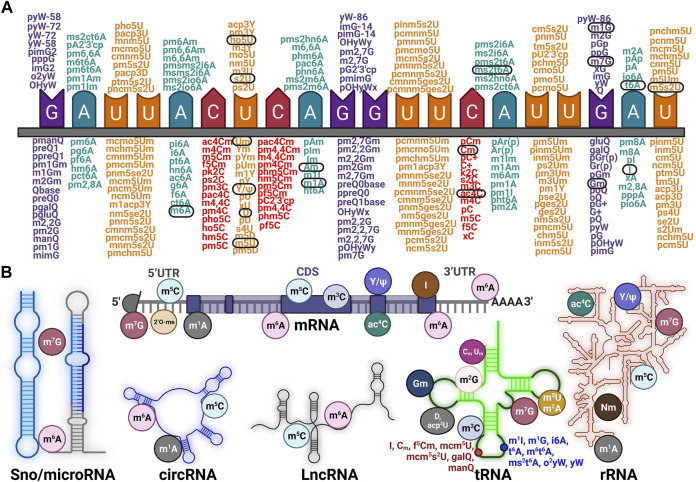
Exhaustive profile for various categories of RNA modifications **(A)**, The authenticated types of RNA modifications according to different single nucleotide base (G- Guanine, C- Cytosine, A- Adenine, U- Uracil) and the conserved ones across species were circled in black. **(B)**, Some kinds of familiar and specific RNA modifications occurred in distinct RNAs like Sno/microRNA, circRNA, LncRNA, tRNA, and rRNA, respectively.

RNA modifications were first detected in highly abundant “infrastructural” RNA (such as rRNAs, tRNAs, snoRNAs, and snRNAs), and were viewed as irreversible decorations that contributed to the structural stability of RNA previously. However, many studies have confirmed that RNA modifications are reversible and that in recent years this has been executed with dynamic modulation of RNA transcription, splicing, localization, decay, and RNA-RBP pattern (Jonkhout et al., 2017; Jiang et al., 2021; Schaefer, 2021). We collected sets of identified RNA modifications that were categorized by their reference nucleotide (G, Adenosine, U, and Cytosine) (Figure 1A). Taking a panoramic view of the situation, RNA modifications, which were highlighted and circled in black, have been established as being associated with diseases (Jonkhout et al., 2017). As for the modifications in RNA of specific categories, this study introduces the principal modifications that occur in mRNA, rRNA, tRNA, LncRNA, circRNA, and Sno/microRNA, respectively (Figure 1B). This illustration indicates that the majority of RNA modifications were mapped to tRNAs, such as Gm, Cm, Um, and yW, with fewer forms identified in mRNA or other RNAs. The dominant type of RNA modification was N1-methyladenosine (m1A), which preferentially enriched in GC content *per se* (Li et al., 2016; Dominissini et al., 2016), 5-methylcytidine (m5C) and was similar to 5 mC in DNA (Squires et al., 2012). The abundance of N6-methyladenosine (m6A) was identified to be 0.1%–0.4% of total adenosine residues and was widespread in mRNA (Dominissini et al., 2012; Meyer et al., 2012), inosine (I) which represented the site-specific conversion of adenosine to inosine (A-to-I) mostly in precursor mRNAs (Levanon et al., 2004). Pseudouridine (*Ψ*) was the most abundantly modification in RNA (Schwartz et al., 2014; Li et al., 2015). N7-methylguanosine (m7G) showed a robust bias installed at the 5′ cap of mRNA during transcription initiation (Zhang et al., 2019). N4-acetylcytidine was focused solely on cytidine mainly within coding sequences (CDS) (Arango et al., 2018) and N6,2′-O-dimethyladenosine (m6A.m.) was mapped to 2′-hydroxyl position of the ribose sugar near m7G (Mauer et al., 2017).

The disruption of gene expression patterns controlled by epigenetics can lead to autoimmune diseases, infection, cancers (Wang et al., 2022), inflammatory, autoimmune diseases and a variety of other diseases (Cui et al., 2022)and m6A methylation plays an important role in both hypertrophic and ischemic heart disease (Kumari et al., 2022), kidney diseases (Ni et al., 2023), such as renal cell carcinoma, acute kidney injury and chronic kidney disease, osteosarcoma (Wu et al., 2022) and other different types of cancers. In a variety of cancers, m6A was found to play different roles, the same regulator has different functions in different cancers, and even different cell lines of the same cancer, and the phenotypes of regulator interventions with similar functions in the same cancer are also different.

Collectively, there have been extensive studies about the modifications of RNA during recent years, with modified nucleotides detected in abundant cellular RNAs and the specifically modified bases might exert various effects in RNA metabolism consisting of structure formation, dynamic stability, splicing, transportation, cellular localization, and translatability (Roundtree et al., 2017). In the next few years, research will likely provide a more systematic and precise atlas for RNA modification and the pathways of diseases.

## 3 Capital RNA modifications associated with cancer hallmarks

The dynamic and elaborate manipulation of reversible RNA modification such as m6A, was previously predominantly dependent on methyltransferases and demethylases. The regulators associated with RNA modification were divided into “Writer (catalyze to effectively induce RNA modification)”, “Erasers (remove the modification in RNA)”, and “Readers/binders (accurately recognize and bind to RNA modification site)”, which could widely influence the steady state and fate of RNAs (Zaccara et al., 2019; Cui et al., 2022). What RNA modification represents in terms of functional and evolutionary significance is still not exhaustive, but it may notably indicate the crossroads between epigenetic regulation and disease (mostly cancer) (Liu and Pan, 2015; Esteve-Puig et al., 2020; Jiang et al., 2021). After this section, we will expound on the influence of the largely dominant RNA modifications (m6A, m5C, and m7G) as examples of tumor progression regarding cancer hallmarks as guidelines.

**FIGURE 2:**
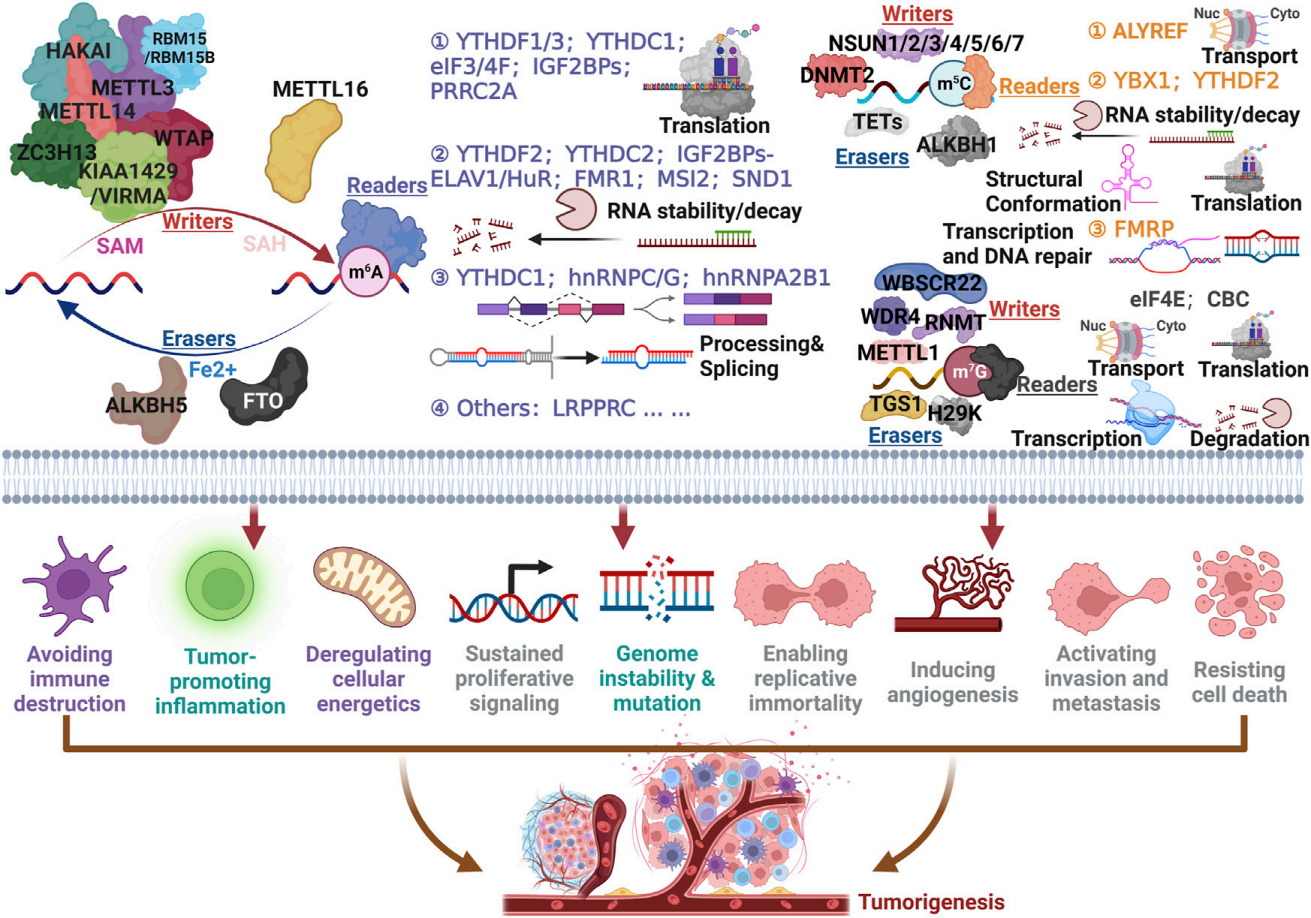
Capital RNA modifications associated with cancer hallmarks. To exemplify the prominent effect of RNA modifications, m6A, m5C, and m7G were designated on account of elucidating accurately the detailed mechanism for modulating tumorigenesis.

Cancer conceptualization aims to distill complex phenotypic and genotypic diversity into a minimally structured set of principles, with the hallmarks continually updated. The latest hallmarks of cancer include avoiding immune destruction, tumor-promoting inflammation, deregulating cellular energetics, sustained proliferative signaling, genome instability, and mutation, enabling replicative immortality, inducing angiogenesis, activating invasion and metastasis, resisting cell death (Hanahan, 2022; Pavlova et al., 2022). Even though a great deal of RNA modifications are catalyzed by enzymes and the fact that the order in which the reactions take place is unknown, the types and roles of RNA-modifying proteins (RMPs) involved in m6A, m5C, and m7G (Figure 2) are summarized below.

First of all, m6A has been well-studied for its role in cancer, and the deposition in RNA are mainly mediated by a methyltransferase (i.e., Writers) complex, including METTL3, METTL14, WTAP, KIAA1459/VIRMA, ZC3H13, HAKAI and RBM15/15B, or METTL16 under existence of SAM. The overwhelming majority of m6A sites occurred at a consensus motif “DRACH” (D = A, G or U; R = A or G; H = A, C or U) (Garcias Morales and Reyes, 2021). Demethylases called “Erasers” contained FTO and ALKBH5, which could effectively remove m6A from RNAs (Roundtree et al., 2017; Esteve-Puig et al., 2020). As regards “Readers”, the wide variety of categories endowed quite different fates for RNA. YTHDF1/3 (Lan et al., 2021; Wiener and Schwartz, 2021), YTHDC1 (Garcias Morales and Reyes, 2021), eIF3/4F (Volpon et al., 2016; Wiener and Schwartz, 2021), IGF2BPs (Huang et al., 2018), and PRRC2A (Wu et al., 2019; Tan et al., 2023) tend to influence the translation efficiency of RNAs. YTHDF2 (Roundtree et al., 2017), YTHDC2 (Roundtree et al., 2017), IGF2BPs-ELAV1/HuR (Huang et al., 2018), FMR1 (Yang et al., 2022; Zhang et al., 2022), MSI2 (Zhu et al., 2022), and SND1 (Baquero-Perez et al., 2019) exerted indispensable roles by accurately dominating the stability and decay of RNAs. Additionally, YTHDC1 (Esteve-Puig et al., 2020), hnRNPC/G (changing the conformation to offer the occupancy motif) (Liu et al., 2017; Zhou et al., 2019) and hnRNPA2B1 (Alarcon et al., 2015) (mainly mediated microRNA mature) significantly contributed to RNA (including mRNA, LncRNA, circRNA and microRNA) processing and splicing. The up-to-date annotated reader, LRPPRC was involved in monitoring translation and the mechanism remains further exploration (Arguello et al., 2017; Wang H. et al., 2023).

When it comes to m5C modification, NSUN1-7 and DNMT2 are ‘Writers’ that have been used to catalyze the methylation that occurred at cytosine, conversely, TETs and ALKBH1 were responsible for the removal of m5C (Nombela et al., 2021; Cui et al., 2022). ALYREF could interact with methylated mRNAs to transport them from nuclei to cytoplasm (Yang et al., 2017). YBX1 and YTHDF2 monitored m5C modified RNAs decay or stability, structural conformation, and translation (Chen et al., 2019; Yang et al., 2019; Wang et al., 2023; Liu et al., 2023). FMRP could assist TET1 to facilitate transcription and DNA repair (Yang et al., 2022).

WBSCR22, WDR4, RNMT, and METTL1 have been shown to be “Writers”, while TGS1 and H29K are “Erasers” for m7G modification (Jonkhout et al., 2017; Liu et al., 2020). Studies have also outlined that eIF4E and CBC affect RNA transcription, transport, translation, and degradation (Izumi et al., 2014; Volpon et al., 2016). Briefly, there is still much to learn about the mechanisms and functions of RNA modification biology, which are just the tip of the iceberg in the study of epigenomes and epitranscriptomes. In the future, oncology therapies may benefit from epitranscriptomic anticancer drugs. It is precisely because RNA modification, especially m6A, plays an indispensable role in the proliferation, invasion, and metastasis of tumor cells and drug resistance, and has great potential for clinical application.

## 4 Therapeutic precis

Treatment for tumors generally comprises surgery, hormonal therapy, Chemo/Target therapy, immunotherapy, radiotherapy, viruses/bacteria (Yanagi et al., 2022), and more recently personalized therapy (Beck et al., 2021; Inoue et al., 2021). The first-line treatment for low-grade hormone receptor-positive metastatic endometrial cancer includes platinum-based chemotherapy and hormonal therapy, and there is no standard follow-up treatment. As a result, new treatment strategies have emerged. Clinical studies of single or combined treatment for endometrial cancer with PARP inhibitors, as well as PD-1 and PD-L1 inhibitors have been reported, which may bring new perspectives to the treatment of metastatic or recurrent endometrial cancer (Karpel et al., 2023; Rowlands et al., 2023).

**FIGURE 3:**
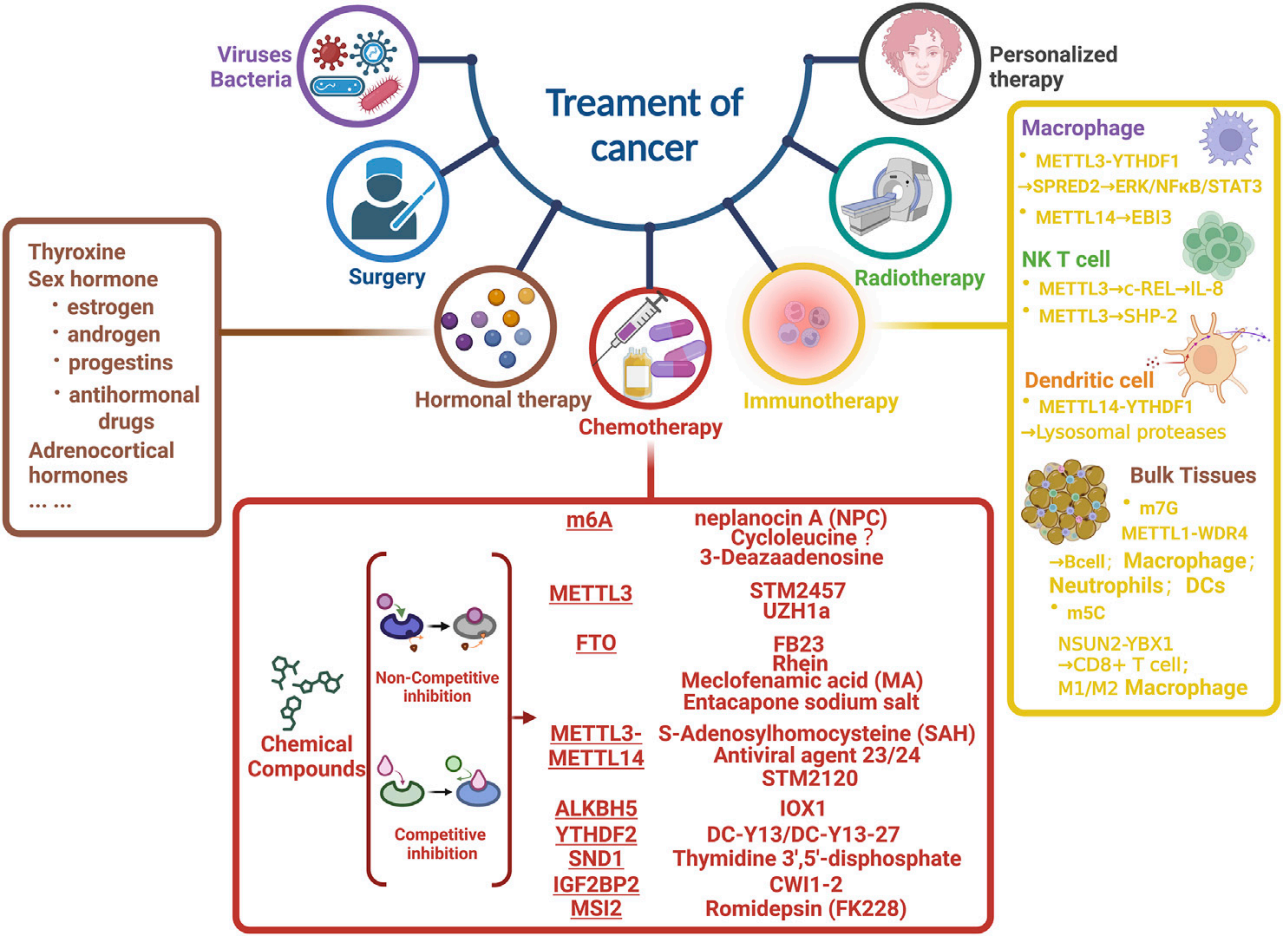
Overview of strategies for m6A related cancer therapy. Focusing on chemotherapy and immunotherapy, we enumerated the main compounds/inhibitors and targets of m6A related protein.

Apart from surgery, hormonal therapy, and radiotherapy, we introduced Chemo/Target therapy and immunotherapy associated with m6A. Above all, we collected the small molecule inhibitors that targeted m6A related proteins, which included (Figure 3):

1 m6A modification, which could be inhibited by neplanocin A (NPC), Cycloleucine, and 3-Deazaadenosine (Cayir, 2022);
2 METTL3, which was effectively repressed by STM2457 (Yankova et al., 2021) and UZH1a (Yanagi et al., 2022);
3 S-Adenosylhomocysteine (SAH), Antiviral agent 23/24 and STM2120 (Yankova et al., 2021) was identified to inhibit the activity of METTL3/14 complex;
4 FB23 (Huang et al., 2019), Rhein, Meclofenamic acid (MA) (Huang et al., 2015), and Entacapone sodium salt could effectively repress FTO;
5 ALKBH5, whose potential inhibitor was IOX1 (Li et al., 2016);
6 DC-Y3 and DC-Y13-27 were for YTHDF2 (Wang et al., 2023);
7 SND1 was corresponding to Thymidine 3′,5′-disphosphate (Jariwala et al., 2017);
8 IGF2BP2, which was restrained by CWI1-2 (Weng et al., 2022);
9 MSI2, whose inhibitor was Romidepsin (FK228) (Cayir, 2022; Zhu et al., 2022).

As for immunotherapy, ablation of METTL3 could effectively impair the YTHDF1 mediated translation of SPRED2 and increase M1/M2 like macrophage, as well as regulatory T cell infiltration into tumours (Yin et al., 2021). Furthermore, METTL3 played a pivotal role in suppressing papillary thyroid carcinoma (PTC) carcinogenesis by synergizing the c-Rel and RelA inactivated nuclear factor B (NF-B) pathways in collaboration with YTHDF2 (He et al., 2021).

Similar to m6A, potential inhibitors of m5C modification indicate that they have an inhibitory effect on tumor cell proliferation with high levels of m5C modification (Fu et al., 2014; Kawarada et al., 2017). Drugs that inhibit m5C modification mainly inhibit m5C modification by acting on m5C regulators, that is, promoting m5C demethylases (Erasers) or inhibiting m5C methyltransferases (Writers) NOP2, NSUN2⁃7^46^, DNMT1, DNMT2 (TRD⁃MT1), DNMT3A and DNMT3B and m5C methylation-binding proteins (Readers) ALYREF, YBX1 and RAD52. Proof-of-concept studies have shown that dysregulated m5C regulators targeted by small molecule inhibitors have the potential for cancer treatment (Garcia-Vilchez et al., 2019; Chen et al., 2020). To date, no m5C inhibitors have been developed (Song et al., 2022). This provides a basis for further research and expansion of the application of these drugs in anti-tumor therapy.

When it comes to the therapeutic precis based on the m6A modification, the effect of m6A on cancer is reflected in the regulation of cancer-related gene expression. There is growing evidence that m6A plays a dual role in cancer (He et al., 2019). On the one hand, m6A regulates the expression of oncogenes or tumor suppressor genes, thereby affecting tumor progression. On the other hand, m6A levels and the expression and activity of m6A enzymes can be regulated, thus affecting the role of m6A in cancer. How m6A influences cancer progression by regulating target genes depends on three factors: 1) whether the target gene acts as a tumor promoter or as a tumor suppressor; 2) abnormal levels of m6A in cancer (depending on changes in expression or activity of “writer” or “erase”); 3) Regulation of target mRNA after methylation (determined by “reader”).

Given the important role of m6A regulatory proteins in a variety of diseases, small molecule inhibitors or agonists that target dysregulated m6A regulatory proteins may be promising candidates for disease treatment, particularly different types of cancer therapies. However, therapeutics targeting m6A modification in cancer are still in their infancy. Future research directions include, but are not limited to, clinical validation of small molecule drugs in cancer patients with abnormal RNA m6A modified protein expression. Therefore, the development of safer and more effective small-molecule inhibitors or agonists of m6A regulatory proteins will help promote the development of RNA-based precision medicine in the future. These scientific findings will contribute to our understanding of the relationship between RNA modifications (m6A, m5C, and m7G) and tumor microenvironment plasticity.

## 5 Systematic profiles of m6A modulation in endometrial cancer

**FIGURE 4:**
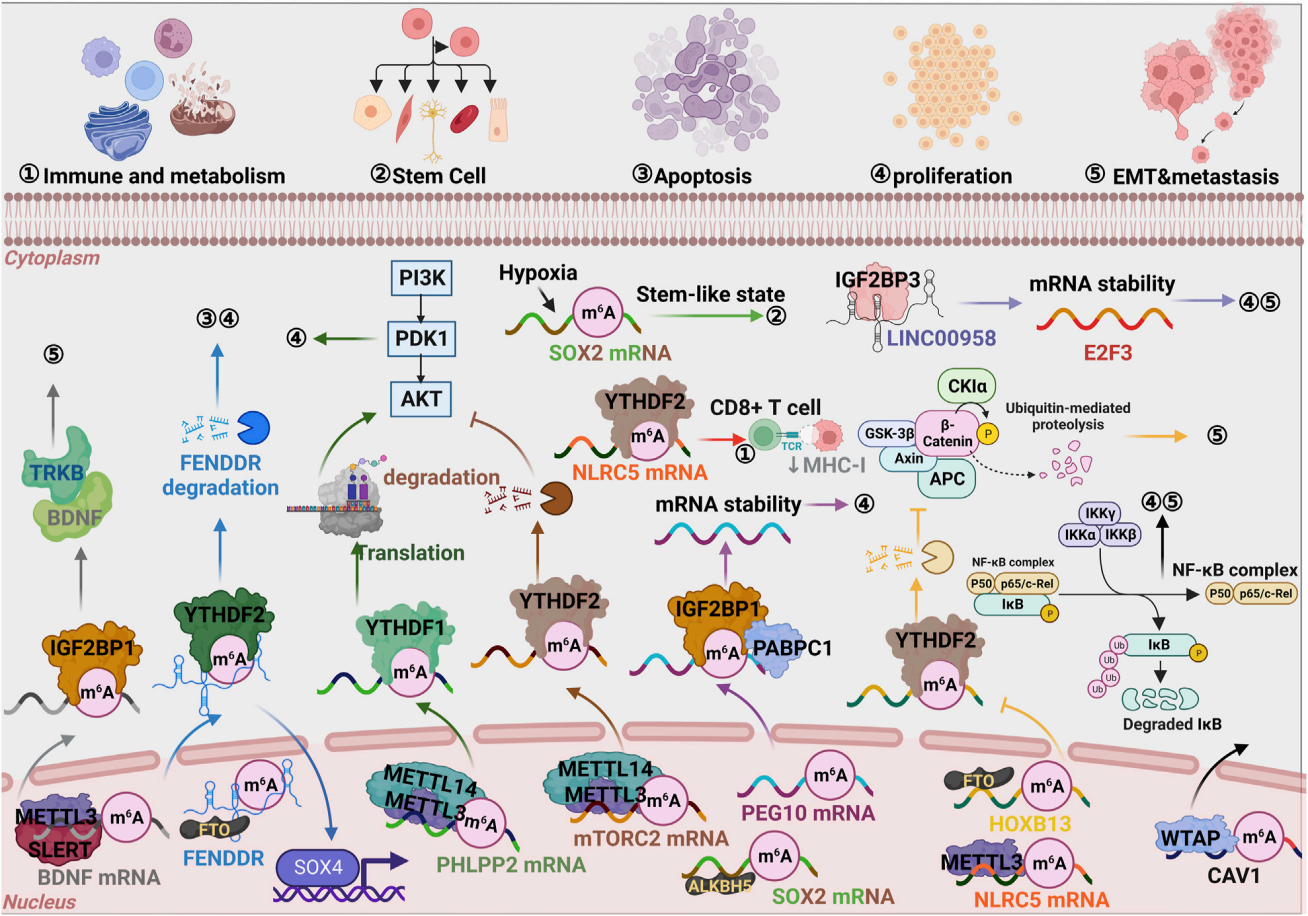
Graphic illustration of m6A monitoring pathway in EC. The diversified modulation axis mediated by core proteins in m6A modification, such as Writers, Erases, and Readers, which ultimately lead to cell proliferation, apoptosis, migration/metastasis, or immune/metabolism during tumor progression.

We systematically summarized the landscape of m6A modification associated regulation pathway in EC (Figure 4). In terms of mechanism, SLERT, as a capable scaffold, could effectively recruit METTL3 and enhance the interaction between the “writer complex” and BDNF mRNA. Accordingly, m6A modified BDNF mRNA was recognized by IGF2BP1 and stabilized to bind TRKB facilitating EC cell metastasis (Tian et al., 2023). This indicates that it could be used as a novel marker to suggest metastasis of endometrial cancer.

In practice, studies showed that upregulated METTL3 was an independent factor in promoting the progression of endometrioid epithelial ovarian cancer (EEOC) through modulating FZD10 and EIF3C, etc (Ma et al., 2020). It was found that overexpression of METTL3 inhibited the proliferation and migration of EC cells and promoted the proliferation of CD8^+^ T cells in the coculture system using EC and CD8^+^ T cells, mechanistically, downregulated METTL3 protects NLRC5 from degradation through m6A modification and YTHDF2 dependent inhibition (Zhan et al., 2023). The conclusion indicated that m6A had a cancer-suppressing effect in EC and would be a novel target for RNA therapy.

LncRNA FENDRR was downregulated and demethylated by FTO in EC cancerous tissues and YTHDF2 was involved in the degradation of FENDRR by m6A recognition, and finally, the expression of SOX4 was elevated to promote cell proliferation (Shen et al., 2021). In addition to m6A modification, LINC00958 also assisted IGF2BP3, significantly enhancing the RNA stability of E2F3, ultimately accelerating EC progression (Wang et al., 2022). Some exciting research in this area described that approximately 70% of endometrial tumors showed reduced m6A methylation, possibly caused by either METTL14 mutation or reduced METTL3 expression. One mechanism was focused on the decreased PHLPP2’s negative regulatory function and the increased mTORC2’s positive regulatory function, as YTHDF1 and YTHDF2 recognized respectively followed by a reduction of m6A methylation (Liu et al., 2018). The model of RNA degradation based on m6A modification, dependent on YTHDF2, was universally accepted. YTHDF2 could not recognize m6A modification as a result of demethylation of HOXB13 mRNA mediated by FTO. With the attenuation of the reduced *HOXB13* mRNA, the Wnt signaling pathway was activated, resulting in EC transfer (Zhang et al., 2021a). The upregulated YTHDF2 could restrain EC progression by accelerating IRS1 degradation and inhibiting IRS1/AKT signaling axis (Hong et al., 2021). This would imply that YTHDF2 plays a role in demethylation to promote tumor malignancy, meaning it could be used as a new target for EC therapy like m6A.

Excitingly, *ß*-estradiol (E2)/estrogen could remarkably induce FTO expression and transfer to nuclei, activate PI3K/AKT and MAPK signal pathways, and participate in enhancing proliferation and invasion of EC through modulating CyclinD1 and MMP2/9 level (Zhang et al., 2012). The high level of ALKBH5, which was another demethylase, under hypoxic conditions facilitated SOX2 mRNA expression by reducing m6A in endometrial cancer stem cells (ECSCs) (Chen et al., 2020). In addition, ALKBH5 enhanced the stability of its mRNA by emethylating IGF1R, thus promoting the proliferation and invasion of EC (Pu et al., 2020). Furthermore, there were many studies on the mechanism by which RNA stability depended on m6A modification. When IGF2BP1 was bound to the m6A site in its 3′UTR and recruited PABPC1 to perform its primary function, the patrol-expressed gene 10 (PEG10) mRNA was stable (Zhang et al., 2021b). Another study also found that oncogenic WTAP enhanced EC proliferation and invasiveness through the caveolin-1(CAV1)/nuclear factor-κB (NF-κB) axis, indicating that CAV1 was identified as a new target for WTAP (Li et al., 2021). Furthermore, many studies have been devoted to the mechanism of RNA stability, which was dependent on m6A modification. Based on the above, the biomarkers that inhibit m6a demethylation (inhibit the “eraser”) or promote m6A methylation (promote the “reader”) play a protective role in EC and could be used as targeted therapeutic targets.

## 6 Network resources

Using bioinformatics analysis, the researchers investigated m6A modifications and their associated genes as potential biomarkers for endometrial cancer. A vital function is played by m6A methylation regulators in the development of endometrial cancer. Age, grade, and risk score were independent risk factors, and a high expression of FTO was associated with poor overall survival, according to both univariate and multivariate Cox regression analyses (Zhang and Yang, 2021). Using the TIMER algorithm to analyze the clinical, sequencing, and copy number variation (CNV) data in The Cancer Genome Atlas (TCGA), which were correlated with m6A regulators, a positive correlation was found between immune cell infiltration and METTL14, ZC3H13, and YTHDC1 level (Jian Ma and Ma, 2021), meanwhile, YTHDC2 performed the same important function in immune infiltration as a prospective biomarker for diagnosis and prognosis (Zhang et al., 2021). Moreover, a potentially useful biomarker for EC prognosis is m7G-related mRNAs. These mRNAs regulated cell cycle progression accompanied by immune cell infiltration, which might lead to UCEC progression (Zhao et al., 2022). Likewise, the findings indicated that the constituents of hazard models relying on the lncRNAs associated with m5C, m7G, or m6A could function as significant intermediaries of the immune milieu and promising prognosis biomarkers with therapeutic response in UCEC (Gu et al., 2022; Sun et al., 2022; Chen et al., 2023; Zhou et al., 2023). Wang et al. outline new insights into CNVs/SNVs in m6A regulatory genes which were associated with a negative impact on patient survival of EC and ultimately found that three genes, *IGF2BP3, KIAA1429,* and *IGF2BP1*, were effective predictors of EC outcomes (Wang et al., 2020). Similarly, Chen et al. selected the risk signature of 8-m6A regulators as the potential predictive prognostic value for EC (Miao et al., 2021).

It is possible to study the epitranscriptome by functionally using RNA modification detection methods and tools. In the wake of the advancement of high-throughput sequencing technologies applied for transcriptome-wide mapping, a number of RNA modification databases have emerged. This is an exciting new research area that encourages additional exploration into the mechanisms and roles of these altered ribonucleotides. Here, we introduced some databases constructed for RNA modification in Table 1, including annotation, site prediction, and functional analysis database. All three primary phylogenetic domains (archaea, bacteria, and eukaryotes) are represented in the RNA Modification Database (RNAMDB) (Cantara et al., 2011). MODOMICS is the most comprehensive source of RNA modification pathways, containing information about the chemical structure of modified nucleosides, their localization in RNA sequences, the pathways of their biosynthesis, and enzymes involved in their synthesis integrated into the model (Boccaletto et al., 2022). Using them, we propose a few suggestions regarding RNA modification databases, with the hope of extending the depth of research in this area.

**TABLE 1:** Some databases constructed for RNA modification.

Database	Website (URL)	Description	Ref
***Comprehensive database of cancer research***			
**UALCAN**	**http://ualcan.path.uab.edu/index.html**	**Providing a platform to explore and analyze the genomic, transcriptomic, or proteomic data through integrating clinical information from TCGA and TCPAC**	Chandrashekar et al. (2022)
**UCSC Xena database**	https://xenabrowser.net/datapages/	**Furnishing high-quality genomics data visualization and genome annotations including detail sequence, CNV, Hi-C heatmap, and RNA-seq data, et.al**	Nassar et al. (2023)
**TIMER package**	**https://cistrome.shinyapps.io/timer/**	**Scores for six types of immune cells (B cell, CD4 T cell, CD8 T cell, Treg, neutrophil, macrophage, and natural killer (NK) cell) were obtained based on mRNA expression data**	Li et al. (2020)
			
***Integrate resources of RNA modification***			
**MODOMICS**	**https://iimcb.genesilico.pl/modomics/**	**A systematic and established database of RNA modification to make the modification site, structure and biosynthetic pathways, location, and associated enzymes accessible to users**	Boccaletto et al. (2022)
**RNAME**	**https://chenweilab.cn/rname/**	**Gathering the experimentally the features of validated enzymes associated with RNA modification, such as structures, domains, locations, and function**	Nie et al. (2022)
**RMVar**	**http://rmvar.renlab.org**	**An updated database of functional variants involved in RNA modifications**	Luo et al. (2021)
**DirectRMDB**	**http://www.rnamd.org/directRMDB/**	**A database of RNA modifications unveiled from direct RNA-seq**	Zhang et al. (2023)
**RMDisease**	**www.xjtlu.edu.cn/biologicalsciences/rmd** **and** **www.rnamd.org/rmdisease2**	**A database of genetic variants that affect RNA modifications, with implications for epitranscriptome pathogenesis**	Chen et al. (2021)
**RM2Target**	**http://rm2target.canceromics.org/**	**A database for writers, erasers, and readers of RNA modifications**	Bao et al. (2023)
**Ariadne**	**http://ariadne.riken.jp/**	**A database search engine for identification and chemical analysis of RNA using tandem mass spectrometry data**	Nakayama et al. (2009)
**RNApathwaysDB**	**http://iimcb.genesilico.pl/rnapathwaysdb**	**A database of RNA maturation and decay/degradation pathways**	Milanowska et al. (2013)
**dreamBase**	**http://rna.sysu.edu.cn/dreamBase**	**The set of DNA modification, RNA regulation, and protein binding pseudogenes unveil new insights into transcriptional regulation**	Zheng et al. (2018)
**RNAWRE**	**http://rnawre.bio2db.com**	**A resource of writers, readers, and erasers of RNA modifications**	Nie et al. (2020)
**RNAInter**	**http://www.rna-society.org/rnainter/**	**RNA interactome repository with increased coverage and annotation**	
**D-lnc**	http://www.jianglab.cn/D-lnc/	**A comprehensive database and analytical platform to dissect the modification of drugs on lncRNA expression**	Jiang et al. (2019)
**GED**	**http://gametsepi.nwsuaflmz.com**	**A manually curated resource for epigenetic modification of gametogenesis**	Bai et al. (2017)
**HAMR software**	**https://github.com/GregoryLab/HAMR**	**High-Throughput Annotation of Modified Ribonucleotides**	Ryvkin et al. (2013)
**RMBase**	**http://mirlab.sysu.edu.cn/rmbase/** **and** **http://rna.sysu.edu.cn/rmbase/**	**A novel resource to interpret the genome-wide landscape of disease related RNA modifications from high-throughput sequencing data**	Sun et al. (2016)
**RNANet**	**https://evryrna.ibisc.univ-evry.fr/evryrna/rnanet**	**An automatically built dual-source dataset integrating homologous sequences and RNA structures**	Becquey et al. (2021)
**POSTAR**	**http://POSTAR.ncrnalab.org**	**A platform for exploring post-transcriptional regulation coordinated by RNA-binding proteins**	Hu et al. (2017)
**EpimiR**	http://bioinfo.hrbmu.edu.cn/EpimiR/	**Establishing the network of epigenetic modifications across multiple species and illuminating the regulatory pathway (including miRNAs)**	Dai et al. (2014)
**TRlnc**	**http://bio.licpathway.net/TRlnc**	**A comprehensive database for human transcriptional regulatory information of lncRNAs, which include typical (super) enhancers and epigenetic regions**	Li et al. (2021b)
**SeqBuster**	**http://estivill_lab.crg.es/seqbuster**	**A bioinformatic tool for the processing and analysis of small RNAs datasets, reveals ubiquitous miRNA modifications in human embryonic cells**	Pantano et al. (2010)
**NcPath**	**http://ncpath.pianlab.cn/** **and** **https://github.com/Marscolono/NcPath/**	**A novel platform for visualization and enrichment analysis of human non-coding RNA and KEGG signaling pathways**	Li et al. (2023)
**AURA**	**http://aura.science.unitn.it**	**Centering on the relationship of trans-factors and UTRs experimentally**	Dassi et al. (2014)
***m6A associated database***			
**m6A-Atlas**	**www.xjtlu.edu.cn/biologicalsciences/atlas**	**A comprehensive knowledgebase for unraveling the m6A epitranscriptome**	Tang et al. (2021)
**RNAMDB**	**http://rna-mdb.cas.albany.edu/RNAmods/**	**A focal point for information pertaining to naturally occurring RNA modifications**	Cantara et al. (2011)
**M6A2Target**	**http://m6a2target.canceromics.org**	**A comprehensive database for targets of m6A writers, erasers, and readers**	Deng et al. (2021)
**REPIC**	**https://repicmod.uchicago.edu/repic**	**An integrated resource of publicly m6A-IP data from various cell lines and tissues**	Liu et al. (2020b)
**M6ADD**	**http://m6add.edbc.org/**	**A comprehensive database of m6A modifications in diseases**	Zhou et al. (2021)
**WHISTLE**	**www.xjtlu.edu.cn/biologicalsciences/whistle** **and** **http://whistle-epitranscriptome.com**	**A predictive framework for acquiring precise landscape of m6A**	Chen et al. (2019b)
**MeT-DB V2.0**	**http://compgenomics.utsa.edu/MeTDB** **and** **www.xjtlu.edu.cn/metdb2**	**A friendly, powerful, and informative web interface to visualize context-specific m6A signaling including peaks or single-base sites**	Liu et al. (2021)
**m6A-TSHub**	**www.xjtlu.edu.cn/biologicalsciences/m6ats**	**A comprehensive online platform, including m6A-TSDB, m6A-TSFinder, m6A-TSVar and m6A-CAVar, to reveal the relationship between m6A modification and genetic mutations**	Song et al. (2022b)
**RNAMethPre**	**http://bioinfo.tsinghua.edu.cn/RNAMethPre/index.html**	**A freely accessible Web Server to predict m6A sites through integrating various characteristics of mRNA upon different treatment conditions**	Xiang et al. (2016)
**SRAMP**	**http://www.cuilab.cn/sramp/**	**Precise prediction tool of mammalian N6-methyladenosine (m6A) sites based on sequence-derived features**	Zhou et al. (2016)
***Resources of other RNA modifications***			
**m5C-Atlas**	**https://www.xjtlu.edu.cn/biologicalsciences/m5c-atlas**	**A comprehensive database for decoding and annotating the m5C**	Ma et al. (2022)
**tRNAmodpred**	**http://genesilico.pl/trnamodpred/**	**Focusing on tRNA modifications, the database provided a computational method for predicting the altered nucleosides of tRNA**	Machnicka et al. (2016)
**tModBase**	**https://www.tmodbase.com/**	**A framework outlining the tRNA modification landscape and its typical features associated with human diseases**	Lei et al. (2023)
**PIANO**	**http://piano.rnamd.com**	**A Web Server for Ψ Identification and Functional Annotation**	Song et al. (2020a)
**Rediportal**	**http://srv00.recas.ba.infn.it/atlas/index.html**	**Collection of novel A-to-I RNA editing events from RNAseq experiments**	Mansi et al. (2021)
**m7GPredictor**	**https://github.com/NWAFU-LiuLab/m7Gpredictor**	**An improved machine learning-based model for predicting internal m7G modifications using sequence properties**	Liu et al. (2020a)
**m7GHub**	**www.xjtlu.edu.cn/biologicalsciences/m7ghub**	**A platform to decipher the location, regulation, disease-associated mutations, and pathogenesis of internal mRNA m7G sites**	Song et al. (2020b)

Bold values are dipicts different types of websites that explain RNA modifications.

Despite advancements in treatment, EC’s incidence and mortality rates continue to rise. A new biomarker and therapeutic target for EC were highlighted in the current study. Future research needs to identify RNA modification regulators and develop a prognostic gene signatures in EC, which are critically important to elucidating cancer pathogenesis and progression.

## 7 Discussion and prospect

At present, more than 150 RNA methylation modifications have been identified as eukaryotic post-transcriptional regulatory markers, and m6A has been well-studied for its role in cancers and the deposition in RNA has been mainly mediated by methyltransferase. With the deepening of research, the role of RNA modifications, especially m6A, in the process of cancer development has attracted more and more attention, but the mechanism of RNA methylation modifications in tumor development needs to be further elucidated. In our review, the types and the roles of RNA methylation modifications in EC are summarized, and the challenges and future directions of RNA methylation modifications in tumor research examined. To facilitate the molecular consideration of further diagnosis and treatment of endometrial cancer, the Cancer Genome Atlas (TCGA) divides endometrial cancer into four different types, including the: 1) DNA polymerase ε (POLE) super mutant type, which had a very high mutation load and a good prognosis; 2) the MicroSatellite Instability (MSI) type (Hrzenjak et al., 2006), with a high mutation load and moderate prognosis; 3) the copy-number low (CNL) type, with low mutation load and moderate prognosis; and 4) the copy-number high (CNH) type, which has a relatively low mutation amount and a poor prognosis. This new classification of endometrial cancer not only provides sufficient prognostic information but also produces subsets of biologically defined ones that may exhibit different responses to specific drugs. For example, POLE hypermutation and deficient mismatch repair (dMMR) endometrial cancers may be more sensitive to PD-1/PD-L1 inhibitor-based immunotherapy because they are associated with high mutational burden and significant immune infiltration. Mismatch repair (MMR) refers to the function of genetic mismatch repair. The MMR gene can express the corresponding MMR protein after transcription and translation, and any loss of expression of MMR protein can cause a base mismatch in the DNA replication process to lose repair function and cause accumulation, resulting in MSI. MSI is divided into microsatellite instability-high (MSI-H), microsatellite instability-low (MSI-L), and stable (MSS). MMR is divided into dMMR and Mismatch Repair Full Function (pMMR). dMMR presents as MSI-H and pMMR as MSI-L or MS-S. Second, CNH endometrial carcinoma is characterized by alterations in the p53 pathway, which was associated with an increased incidence of homologous recombination deficiency (HRD), and in general, HRD tumors might respond to PARP inhibitors (Xiong et al., 2006; Zhou et al., 2007; Tao and Freudenheim, 2010; Zhang et al., 2012). Since RNA can be detected in serum or plasma *in vivo*, it can be used for early diagnosis, assessment of efficient treatment, and prognosis prediction of EC. It is also expected to provide new ideas and new targets for the pathogenesis and therapy of EC (Pavlova et al., 2022; Xu et al., 2022). Over the past few years, it has been possible to identify the chemical basis and multiple functions of m6A RNA methylation due to the availability of highly specific antibodies and the availability of high-throughput sequencing techniques. With the rapid development of m6A crosslinking-immunoprecipitation and RNA-seq technology, m6A has been shown to be involved in the development of a variety of malignancies. This will enable the targeting of m6A-related enzymes or m6A-dependent pathways, providing an important scientific basis for the targeted treatment of human cancer with m6A.

Although the role of m6A in cancer has gradually been revealed, many challenges remain. Firstly, the mechanism of m6A regulators in tumors is largely unknown, such as the role and mechanism of “Readers” in cancer in m6A methylation modification is still a big gap; Secondly, although many studies have shown that m6A-related regulators and pathways could be used as new targets in cancer treatment, there is a lack of certain clinical practice, and m6A can affect the expression of genes in many aspects. That is to say, its side effects cannot be ignored. The coming mission will aim to deeply explore the molecular mechanism of “Writers”, “Erasers” and “Readers” in m6A modification involved in the regulation of tumorigenesis, as well as evaluate the correlation between m6A and cancer in combination with clinical data. Therefore, strengthening our understanding of tumor malignant transformation, ultimately, will be conducive to seeking and designing novel prospective targets for cancer therapy soon.
